# Mitochondrial and lysosomal biogenesis are activated following PINK1/parkin‐mediated mitophagy

**DOI:** 10.1111/jnc.13412

**Published:** 2015-11-24

**Authors:** Davor Ivankovic, Kai‐Yin Chau, Anthony H. V. Schapira, Matthew E. Gegg

**Affiliations:** ^1^ Department of Clinical NeuroscienceUCL Institute of NeurologyLondonUK

**Keywords:** lysosomes, mitophagy, Nrf2, Parkinson's disease, PINK1, TFEB

## Abstract

Impairment of the autophagy–lysosome pathway is implicated with the changes in α‐synuclein and mitochondrial dysfunction observed in Parkinson's disease (PD). Damaged mitochondria accumulate PINK1, which then recruits parkin, resulting in ubiquitination of mitochondrial proteins. These can then be bound by the autophagic proteins p62/SQSTM1 and LC3, resulting in degradation of mitochondria by mitophagy. Mutations in *PINK1* and *parkin* genes are a cause of familial PD. We found a significant increase in the expression of p62/SQSTM1 mRNA and protein following mitophagy induction in human neuroblastoma SH‐SY5Y cells. p62 protein not only accumulated on mitochondria, but was also greatly increased in the cytosol. Increased p62/SQSMT1 expression was prevented in PINK1 knock‐down cells, suggesting increased p62 expression was a consequence of mitophagy induction. The transcription factors Nrf2 and TFEB, which play roles in mitochondrial and lysosomal biogenesis, respectively, can regulate p62/SQSMT1. We report that both Nrf2 and TFEB translocate to the nucleus following mitophagy induction and that the increase in p62 mRNA levels was significantly impaired in cells with Nrf2 or TFEB knockdown. TFEB translocation also increased expression of itself and lysosomal proteins such as glucocerebrosidase and cathepsin D following mitophagy induction. We also report that cells with increased TFEB protein have significantly higher PGC‐1α mRNA levels, a regulator of mitochondrial biogenesis, resulting in increased mitochondrial content. Our data suggests that TFEB is activated following mitophagy to maintain autophagy–lysosome pathway and mitochondrial biogenesis. Therefore, strategies to increase TFEB may improve both the clearance of α‐synuclein and mitochondrial dysfunction in PD.

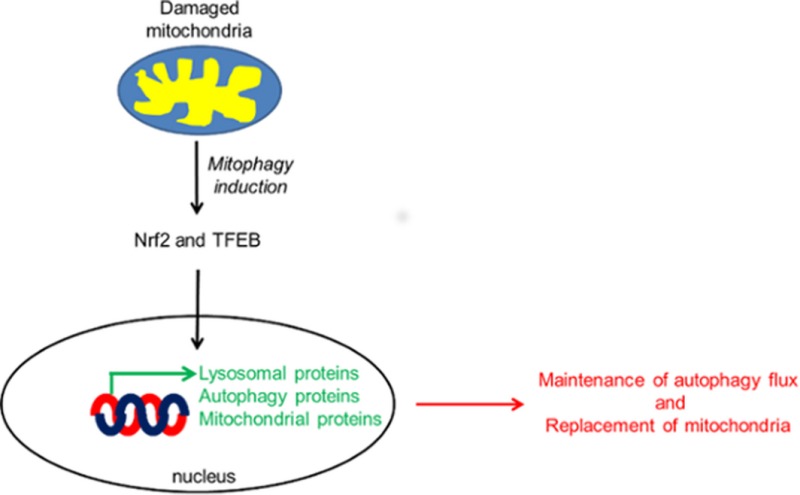

Damaged mitochondria are degraded by the autophagy–lysosome pathway and is termed mitophagy. Following mitophagy induction, the transcription factors Nrf2 and TFEB translocate to the nucleus, inducing the transcription of genes encoding for autophagic proteins such as p62, as well as lysosomal and mitochondrial proteins. We propose that these events maintain autophagic flux, replenish lysosomes and replace mitochondria.

**Abbreviations used**
ALP: autophagy–lysosome pathway
AP: autophagosomes
ARE: antioxidant response element
Baf A1: bafilomycin A1
BSA: bovine serum albumin
CCCP: carbonyl cyanide m‐chlorophenylhydrazone
CLEAR: coordinated lysosomal enhancement and regulation
CMA: chaperone mediated autophagy
CS: citrate synthase
DAPI: 4′,6‐diamidino‐2‐phenylindole
DTT: dithiothreitol
GCase: glucocerebrosidase
HEX: β‐hexosaminidase
KD: knockdown
KO: knockout
MEFs: mouse embryonic fibroblasts
MFN: mitofusin
NQO1: NAD(P)H dehydrogenase quinone 1
OMM: outer mitochondrial membrane
PBS: phosphate buffered saline
PD: Parkinson's disease
TFEB: transcription factor EB

The function of mitochondria and lysosomes decreases with age (Terman *et al*. 2010; Nixon 2013). Age is the greatest risk factor for developing Parkinson's disease (PD), and the dysfunction of mitochondria and the autophagy–lysosome pathway (ALP) are both implicated in the pathogenesis of PD (Schapira and Gegg 2011; Nixon 2013). The failure of macroautophagy and chaperone‐mediated autophagy, which control the degradation of damaged mitochondria and α‐synuclein are considered particularly important in the bioenergetic dysfunction and protein accumulation/aggregation observed in PD (Narendra *et al*. 2008, 2010b; Alvarez‐Erviti *et al*. 2010; Dehay *et al*. 2010; Vives‐Bauza *et al*. 2010).

Mutations in *PINK1* and *PARK2 (parkin)* genes have been identified as causes of autosomal recessive PD (Kitada *et al*. 1998; Valente *et al*. 2004). Loss of PINK1 or parkin function results in mitochondrial dysfunction (Schapira and Gegg 2011). Initial work with PINK1 and parkin in *Drosophila* models suggested that these proteins function in the same signalling pathway to maintain mitochondrial function (Clark *et al*. 2006; Park *et al*. 2006). Consequently, it has been shown that in mammalian cells, chemical induction of mitophagy by use of the uncoupler carbonyl cyanide m‐chlorophenylhydrazone (CCCP) causes stabilisation of PINK1 on the outer mitochondrial membrane. PINK1 on the outer mitochondrial membrane in turn recruits parkin in a kinase‐dependent manner (Matsuda *et al*. 2010; Narendra *et al*. 2010b; Vives‐Bauza *et al*. 2010; Kondapalli *et al*. 2012). Parkin is an E3 ligase, which following recruitment to depolarised mitochondria, ubiquitinates many mitochondrial proteins. Ubiquitinated substrates include VDAC1, mitofusins and TOM20 (Gegg *et al*. 2010; Geisler *et al*. 2010; Tanaka *et al*. 2010; Chan *et al*. 2011; Sarraf *et al*. 2013). Ubiquitination of these substrates primes mitochondria for recruitment to phagophores that then mature in to autophagosomes (AP), which are double membrane vesicles in which cargo destined for degradation are sequestered (Nixon 2013). AP then fuse with lysosomes resulting in degradation of dysfunctional mitochondria. Ubiquitinated mitochondrial proteins can either bind directly to the phagophore by binding the autophagic adaptor protein LC3‐II, which is embedded in the phagophore membrane or via p62 (SQSTM1). P62 contains a LC3 interacting domain, and facilitates the recruitment of damaged mitochondria to the phagophore by binding to LC3‐II (Ding *et al*. 2010; Geisler *et al*. 2010). The phosphorylation of p62 has been proposed to initiate the binding of p62 to ubiquitinated proteins during autophagy and mitophagy (Ichimura *et al*. 2013). During mitophagy, mitochondria destined for degradation cluster next to the nucleus (Lee *et al*. 2010; Narendra *et al*. 2010a). The interaction between mitochondria, p62, HDAC6 and cortactin has been implicated in mediating this perinuclear localisation of mitochondria (Lee *et al*. 2010; Yan *et al*. 2013). Furthermore, it has been proposed that K63‐linked polyubiquitination of mitochondrial proteins by parkin recruits p62, which then polymerises via its PB1 domain, causing the mitochondria to aggregate next to the nucleus prior to recruitment in to AP (Narendra *et al*. 2010a; Okatsu *et al*. 2010).

In addition to recruitment of mitochondria to AP, the ubiquitination of mitochondrial proteins also prevents ‘primed’ mitochondria from refusing with the ‘healthy’ mitochondrial population by rapidly degrading pro‐fusion proteins including mitofusins via the proteasome (Tanaka *et al*. 2010; Gegg and Schapira 2011). The mitochondrial dysfunction associated with aberrant PINK or parkin function is likely, at least in part, to increase the accumulation of damaged mitochondria. Indeed, impairment of oxidative phosphorylation in PINK1‐deficient SH‐SY5Y neuroblastoma cells was reversed by over‐expressing parkin and restoring autophagic flux (Gegg *et al*. 2010). However, it should be noted that constitutive knockdown (KD) of PINK1 expression in SH‐SY5Y cells has been reported to activate mitophagy in the absence of CCCP (Dagda *et al*. 2009).

In order to maintain mitochondrial function in cells, the removal of damaged mitochondria by mitophagy needs to be carefully regulated and counter‐balanced by the biogenesis of mitochondria (Zhu *et al*. 2013). This is especially critical in neurons that are very dependent on mitochondrial ATP synthesis (Van Laar and Berman 2013). Transcription of PINK1 has been reported to be significantly increased 24 h after induction of mitophagy by CCCP (Gómez‐Sánchez *et al*. 2014). This is of particular interest as PINK1 has been linked with mitochondrial biogenesis and the regulation of mitochondrial DNA levels (Gegg *et al*. 2009; Tufi *et al*. 2014). The PGC‐1 family of co‐activators (PGC‐1α, PGC‐1β and PRC) play pivotal roles in cellular metabolism and mitochondrial biogenesis via their interactions with several transcription factors such as Nrf1, Nrf2 and cAMP‐response element binding protein (Scarpulla 2011; Zhu *et al*. 2013).

The biogenesis of lysosomes and transcription of autophagic proteins is also tightly regulated, and a coordinated lysosomal enhancement and regulation network of genes has been identified (Sardiello *et al*. 2009). Many lysosomal genes have coordinated lysosomal enhancement and regulation sites in their promoters and are activated by the basic‐loop‐helix‐loop family of transcription factors such as transcription factor EB (TFEB) and MITF (Sardiello *et al*. 2009; Settembre and Ballabio 2014). The activation of TFEB has been suggested as a treatment for PD, since increased expression of this transcription factor protects against α‐synuclein and 1‐methyl‐4‐phenyl‐1,2,3,6‐tetrahydropyridine mediated dopaminergic cell death (Dehay *et al*. 2010; Decressac *et al*. 2013).

We report that induction of mitophagy in SH‐SY5Y cells results in increased transcription and translation of the important autophagic protein p62/SQSMT1 and key lysosomal enzymes such as glucocerebrosidase (GCase). The transcription factors Nrf2 and TFEB were found to translocate to the nucleus upon mitophagy induction, resulting in transcription of downstream targets. Our results highlight the integration of the mitochondrial and lysosomal biogenesis pathways and have important implications for cell homeostasis, bioenergetics and protein turnover.

## Materials and methods

### Cell culture

The human neuroblastoma SH‐SY5Y cell line (obtained from European Collection of Cell Cultures) was cultured in 1:1 (v/v) Dulbecco's modified Eagle's medium:F12 (Ham) media supplemented with 10% fetal bovine serum, 1 mM pyruvate and penicillin–streptomycin. SH‐SY5Y cells with constitutive KD of PINK1 were generated using HuSH 29mer shRNA constructs (pRS plasmid; Origene, Rockville, MD, USA) against PINK1 (GCTGTGTATGAAGCCACCATGCCTACATT) or scrambled negative control shRNA. SH‐SY5Y cells were transfected with the constructs and grown under puromycin selection in media described above. Human TFEB with a Myc‐DDK tag (pCMV6‐Entry; Origene) or PGC‐1α with a hemagglutinin tag (pCDNA3.1; kind gift from Dr Anastasia Kralli, The Scripps Research Institute, USA) were transfected in to SH‐SY5Y cells and stable expressing clones selected using G418 (400 μg/mL media). All genetic modifications of cells were approved by the University College London.

### Transient transfection of SH‐SY5Y cells with siRNA

SH‐SY5Y cells (1.8 × 10^5^ cells/mL) were transfected with 40 nM p62 siRNA (sense, CUUCCGAAUCUACAUUAAAtt; antisense, UUUAAUGUAGAUUCGGAAGat; Ambion, Austin, TX, USA) or 25 nM TFEB siRNA (sense, GCCUGGAGAUGACCAACAATT; antisense, UUGUUGGUCAUCUCCAGGCGG; Qiagen, Valencia, CA, USA) or 100 nM Nrf2 siRNA (sense, GAAUGGUCCUAAAACACCAtt; antisense, UGGUGUUUUAGGACCAUUCtg; Ambion) or scrambled control siRNA (Ambion) using HiPerfect transfection reagent (Qiagen). Cells were transfected with siRNA for 3 days.

### Citrate synthase activity

Following treatment with 10 μM CCCP, cells were harvested, washed once with phosphate buffered saline (PBS) and lysed in 0.25% (v/v) Triton X‐100 in PBS. Debris was removed by centrifugation and citrate synthase (CS) activity measured by the following the oxidation of 5,5′‐Dithiobis(2‐nitrobenzoic acid) in a spectrophotometer (absorbance at 412 nm) over time at 30°C in the presence of acetyl co‐enzyme A and oxaloacetate (Gegg *et al*. 2010). Protein concentration in the same aliquot was measured using the bicinchoninic acid protein assay (Pierce, Rockford, IL, USA) and enzyme activity expressed as nmol/min/mg protein.

### Western blotting

SH‐SY5Y cells were harvested with trypsin and lysed on ice in 1% (v/v) Triton X‐100 in PBS supplemented with protease and phosphatase inhibitors or 0.1% (v/v) sodium dodecyl sulphate (SDS), 10 mM Tris pH 7.4, 150 mM NaCl supplemented with DNase and protease/phosphatase inhibitors. Protein lysates (20 μg) were resolved by sodium dodecyl sulphate–polyacrylamide gel electrophoresis and transferred to Hybond P (GE Healthcare, Little Chalfont, UK). Blots were probed with antibodies against Nrf2 (ab31163; Abcam, Cambridge, UK), p62 (610833; BD Biosciences, Oxford, UK), prohibitin 1 (2426; Cell Signaling Technology, Beverly, MA, USA), cytochrome oxidase (COX) subunit IV (ab14744; Abcam), LC3B (2775; Cell Signaling Technology), cathepsin D (ab6313; Abcam), TOM20 (FL‐145; Santa Cruz Biotechnology, Santa Cruz, CA, USA), TFEB (ab2636; Abcam), DDK (clone 4C5; Origene), lamin A (ab26300; Abcam), PINK1 (BC100‐494; Novus Biologicals, Cambridge, UK), PGC‐1α (NBP1‐04676; Novus Biologicals) or β‐actin (ab82618; Abcam). Bands were detected with respective horse radish peroxidase‐linked secondary antibodies (Dako, Carpinteria, CA, USA) and enhanced chemiluminescence (Pierce). Density of bands was determined using ImageJ software (NIH, Bethesda, MD, USA) or Image Lab software 5.1 (Bio‐Rad Laboratories, Hercules, CA, USA).

### Immunofluorescence

Following treatment with CCCP, SH‐SY5Y cells on coverslips were fixed in 3.7% paraformaldehyde. For detection of p62, cells were permeabilised with glacial methanol, blocked in 2% goat serum in PBS/0.1% Triton X‐100 and incubated with antibodies against p62 (610833; BD Biosciences) and TOM20 (FL‐145; Santa Cruz Biotechnology). For detection of Nrf2 or TFEB, cells were permeabilised with PBS/0.1% Triton X‐100, blocked with 2% goat serum and 2% bovine serum albumin in PBS/0.1% Triton X‐100, and incubated with Nrf2 antibody (ab31163; Abcam) or DDK antibody (see above) overnight at 4°C. Respective fluorescent secondary antibodies (alexa fluor 488, 594; Invitrogen, Carlsbad, CA, USA) were used for detection and coverslips mounted in citifluor containing 4′,6‐diamidino‐2‐phenylindole.

### Quantitative real‐time PCR

Following treatment, RNA was extracted from SH‐SY5Y cells using RNeasy kit (Qiagen). RNA was converted to cDNA (Primer Design, Southampton, UK) and relative mRNA levels were measured using SYBERgreen (Applied Biosystems, Paisley, UK). Relative expression of PGC‐1α, NAD(P)H dehydrogenase quinone 1 (NQO1), p62 and PINK1 mRNA was measured with Power SYBRgreen kit (Applied Biosystems) using a STEP One PCR machine (Applied Biosystems). β‐actin mRNA levels were used to normalise data. Primers are listed in Table 1. Relative expression was calculated using the ΔC_T_ method.

**Table 1:** Primers for quantitative real‐time PCR

Target	Sequence	Annealing temperature (°C)
β‐actin	5′‐TCTACAATGAGCTGCGTGTG‐3′ 5′‐GGTGAGGATCTTCATGAGGT‐3′	58
GCase	5′‐TGCTGCTCTCAACATCCTTGCC‐3′ 5′‐TAGGTGCGGATGGAGAAGTCAA‐3′	58
NQO1	5′‐CAGCTCACCGAGAGCCTAGT‐3′ 5′‐GAGTGAGCCAGTACGATCAGTG‐3′	62
p62	5′‐CAGAGAAGCCCATGGACAG‐3′ 5′‐AGCTGCCTTGTACCCACATC‐3′	58
PGC‐1α	5′‐CAGAGAACAGAAACAGCAGCA‐3′ 5′‐TGGGGTCAGAGGAAGAGATAAA‐3′	58
PINK1	5′‐GGACGCTGTTCCTCGTTA‐3′ 5′‐ATCTGCGATCACCAGCCA‐3′	58
TFEB	5′‐CCAGAAGCGAGAGCTCACAGAT‐3′ 5′‐TGTGATTGTCTTTCTTCTGCCG‐3′	60

GCase, glucocerebrosidase; NQO1, NAD(P)H dehydrogenase quinone 1; TFEB, transcription factor EB.

### Measurement of glucocerebrosidase and β‐hexosaminidase activity

Following treatment, SH‐SY5Y cells were lysed in 1% (v/v) Triton X‐100 in PBS and GCase activity determined in samples (20 μg protein) by hydrolysis of 5 mM 4‐methylumbelliferyl‐β‐d‐glucopyranoside in McIIvaine buffer (pH 5.4) in the presence of sodium taurocholate at 37°C for 1 h (Gegg *et al*. 2012). The reaction was stopped by addition of 0.25M glycine (pH 10.4) and 4‐methylumbelliferone fluorescence measured at excitation 360 nm, emission 460 nm.

β‐hexosaminidase (HEX) was assayed in above lysates (2 μg protein) using the fluorogenic substrate 4‐methylumbelliferyl‐2‐acetoamido‐2‐deoxy‐6‐sulfo‐β‐d‐glucopyransoside (2 mM) in sodium citrate buffer (pH 4.2) at 37°C for 30 min. The reaction was stopped by addition of 0.25M glycine (pH 10.4) and 4‐methylumbelliferone fluorescence measured at excitation 360 nm, emission 460 nm.

### Mitochondrial isolation

SH‐SY5Y cells were treated with 10 μM CCCP for 18 h and cytosolic and mitochondria‐enriched fractions prepared as previously described (Gegg *et al*. 2010). Equal amounts of protein (20 μg) from each fraction were measured by western blotting. Given the differing total volumes of the two fractions (mitochondria 250 μL; cytosol 4000 μL), this equates to approximately 0.5% and 4% of total cytosolic and mitochondria‐enriched fractions respectively. Extrapolation from western blot analysis of β‐tubulin band density indicates that the total amount of β‐tubulin in the mitochondria‐enriched fraction was 3.5 ± 1.5% (*n* = 3) of the cytosolic fraction.

### Nuclear isolation

For the detection of Nrf2 and PGC‐1α, cytosolic and nuclear fractions were prepared using the EpiQuik Nuclear Extraction Kit II (Epigentek, Farmingdale, NY, USA). SH‐SY5Y cells and PGC‐1α over‐expressing cells (~7 × 10^6^ cells) were harvested by trypsinisation. For the detection of TFEB, nuclear and cytoplasmic fractions were prepared from ~2 × 10^6^ cells by scraping in to a hypotonic buffer (10 mM Hepes (pH 7.9), 10 mM KCl, 0.1 mM EDTA, 0.1 mM EGTA, 1 mM dithiothreitol, 0.15% NP‐40), and homogenising with 20 strokes of a Dounce homogenizer. An aliquot (100 μL; 25% total) was reserved for total lysate and was supplemented with 1% (w/v) SDS and 10 units of DNase (Promega, Madison, WI, USA). Remainder was centrifuged at 17 000 *g* for 5 min at 4°C. Supernatant (cytosol) was removed and the nuclear pellet resuspended in 200 μL high salt buffer (20 mM Hepes, 400 mM NaCl, 1 mM EDTA, 1 mM EGTA, 1 mM dithiothreitol, 0.5% (v/v) NP‐40) and solubilised with SDS (1% (w/w) final and 10 U DNase (Roczniak‐Ferguson *et al*. 2012). Protein concentration of each fraction was measured using bicinchoninic acid protein assay kit.

### Statistical analyses

Data are expressed as mean ± SEM and statistical significance between groups determined by one‐tailed *t*‐test or one‐way anova followed by the Tukey HSD test.

## Results

### Expression of p62/SQSTM1 increased following CCCP‐induced mitophagy

**Figure 1:**
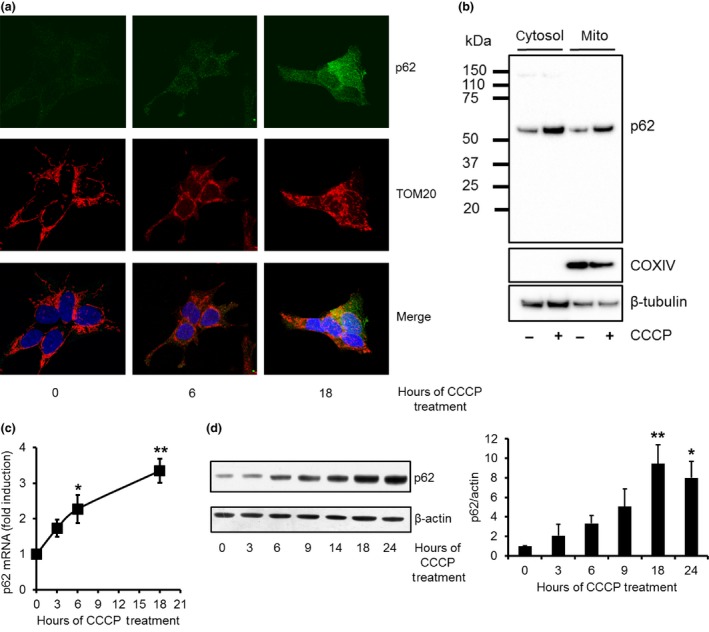
p62 protein expression is increased following carbonyl cyanide m‐chlorophenylhydrazone (CCCP) treatment. (a) SH‐SY5Y cells were treated with 10 μM CCCP for 6 or 18 h and p62 (green) and the mitochondrial protein TOM20 (red) were detected by immunofluorescence. Nuclei were counter‐stained with 4′,6‐diamidino‐2‐phenylindole (blue) (b) SH‐SY5Y cells were treated with 10 μM CCP for 18 h, homogenised and cytosolic and mitochondrial enriched fractions prepared. Fractions (20 μg protein) were analysed by western blot for p62 protein. Fractions were also probed for the mitochondrial protein cytochrome oxidase (COX) subunit IV and the cytosolic marker β‐tubulin. (c) SH‐SY5Y cells were treated with 10 μM CCCP and p62 mRNA levels measured by qPCR (*n* = 7) or (d) p62 protein levels by western blotting (*n* = 6). *p <* *0.05 versus 0 h; **p <* *0.01 versus 0 h.

The autophagic protein p62/SQSTM1 is known to co‐localise with depolarised mitochondria in order to facilitate their recruitment to AP, which is then degraded by macroautophagy. Treatment of the human neuroblastoma SH‐SY5Y cell line with the mitochondrial uncoupler CCCP (10 μM) for 18 h resulted in co‐localisation of p62 with the mitochondrial outer membrane protein TOM20 (Fig. 1a). During this period, p62 protein levels also appeared to increase in the cytoplasm. Subcellular fractionation also indicated increased p62 protein in mitochondria‐enriched fractions (Fig. 1b). We also observed a notable increase in p62 protein expression in the cytosolic fraction after 18 h of CCCP treatment (Fig. 1b). Analysis of p62 mRNA levels by quantitative real‐time PCR and western blotting for p62 protein levels confirmed that p62 expression was increased following CCCP treatment (Fig. 1c and d).

**Figure 2:**
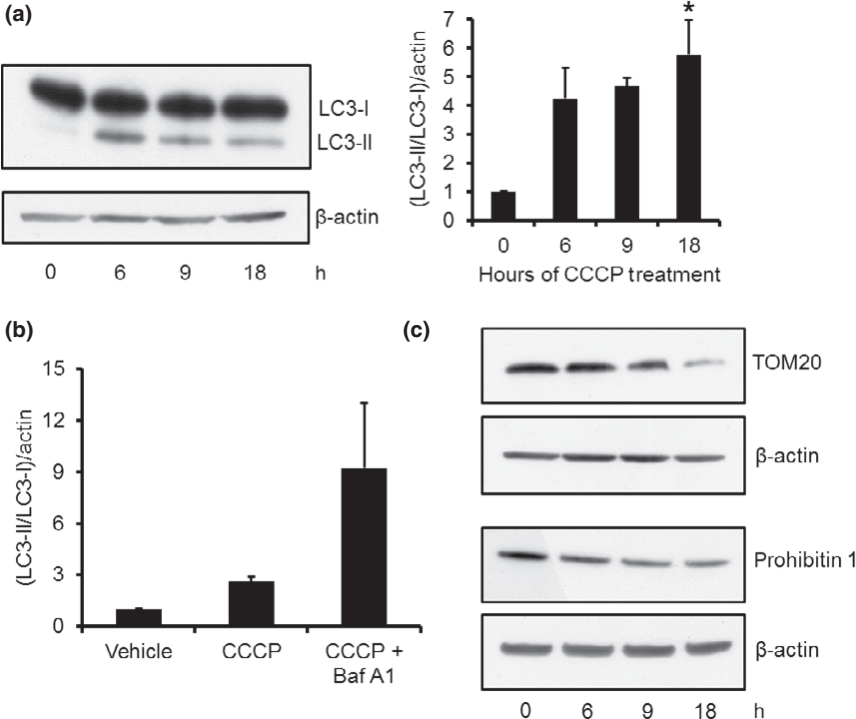
Macroautophagy flux is increased and mitochondrial content decreased following carbonyl cyanide m‐chlorophenylhydrazone (CCCP) treatment. (a) SH‐SY5Y cells were treated with 10 μΜ CCCP and LC3‐II protein levels measured by western blot. The ratio of (LC3‐II/LC3‐I)/β‐actin was calculated (*n* = 4). (b) Macroautophagy flux was measured by treating SH‐SY5Y cells with vehicle, 10 μΜ CCCP, or 10 μΜ CCCP + 100 nM bafilomycin A1 for 6 h and LC3‐II protein levels measured by western blotting (*n* = 4). Data expressed as (LC3‐II/LC3‐I)/β‐actin. (c) The decrease in mitochondrial content was measured by western blot after 6, 9 or 18 h of CCCP treatment using antibodies against the mitochondrial proteins TOM20 and prohibitin 1. *p <* *0.05 versus 0 h.

To further prove that increased p62 protein levels were because of *de novo* expression, rather than inhibition of macroautophagy/mitophagy, we measured LC3‐II protein levels by western blot, a marker of AP number. LC3‐II protein levels were increased following CCCP treatment suggesting increased formation of AP (Fig. 2a). Treatment with bafilomycin A1 (Baf A1), which inhibits fusion of AP with lysosomes, further increased LC3‐II levels, indicating that the increase in AP number following CCCP treatment was because of increased macroautophagy flux (Fig. 2b). Furthermore, the mitochondrial content of cells was decreased following CCCP treatment. The protein levels of TOM20 (outer membrane) and prohibitin 1 (inner membrane) were diminished following 18 h of CCCP treatment (Fig. 2c). These two proteins have been shown by us and others to be ubiquitinated and degraded following CCCP‐induced PINK1/parkin‐mediated mitophagy (Chan *et al*. 2011; Sarraf *et al*. 2013; Gómez‐Sánchez *et al*. 2014).

### p62 protein increased following induction of mitophagy but not starvation‐induced macroautophagy

**Figure 3:**
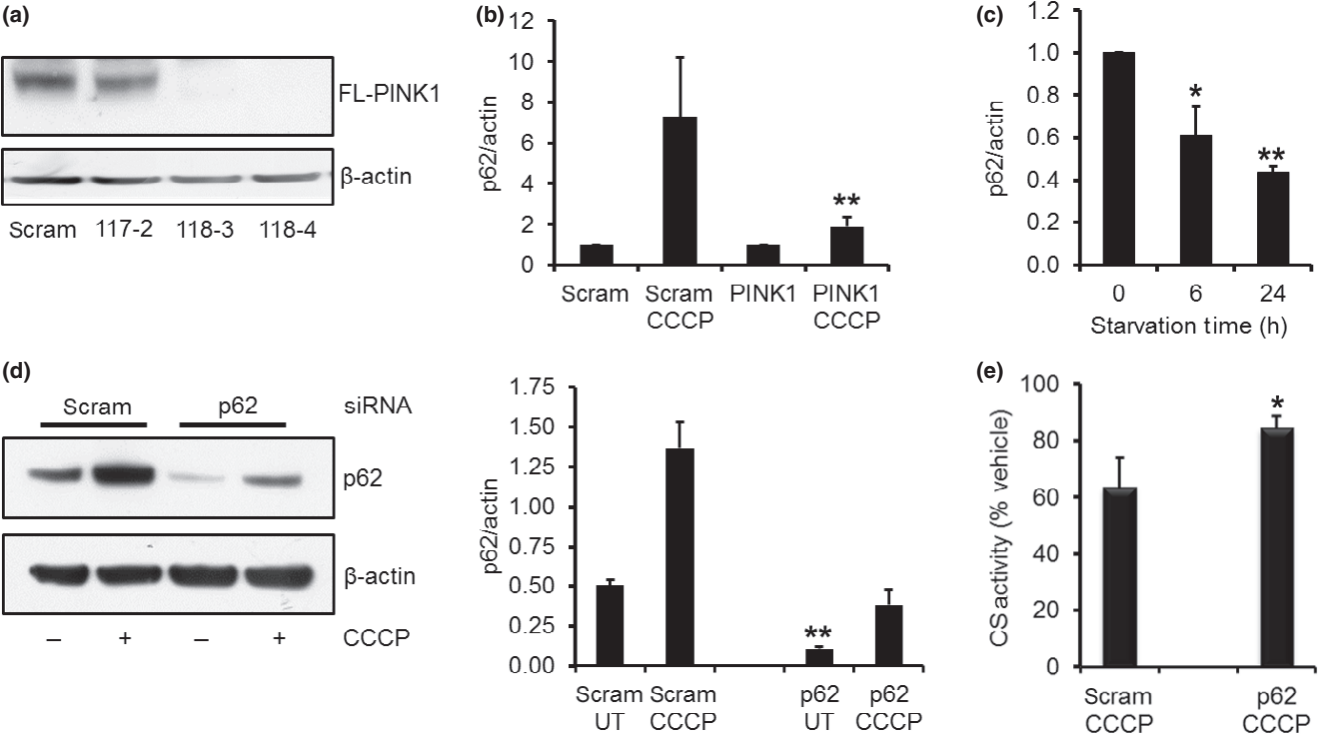
Increased p62 expression is prevented in PINK1 knock‐down cells. (a) SH‐SY5Y cells expressing scrambled shRNA or PINK1 shRNA (117‐2, 118‐3, and 118‐4) were treated with 10 μM carbonyl cyanide m‐chlorophenylhydrazone (CCCP) for 24 h and full‐length (FL) PINK1 protein levels measured by western blot. No accumulation of FL PINK1 was observed in 118‐3 and 118‐4 PINK1 shRNA clones. (b) SH‐SY5Y cells constitutively expressing scrambled (scram) or PINK1 shRNA (clone 118‐3) were treated with vehicle or 10 μM CCCP for 24 h and p62 protein expression measured by western blotting. **p <* *0.05 versus scram CCCP;* n* = 9. (c) SH‐SY5Y cells were incubated in serum‐free culture media for up to 24 h to induce starvation‐mediated macroautophagy. p62 protein levels were measured by western blotting. *p <* *0.05 versus 0 h; **p <* *0.01 versus 0 h; *n* = 4. (d) SH‐SY5Y cells were transfected with scrambled (scram) or p62 siRNA for 72 h. For the last 24 h, cells were treated with vehicle (ethanol) or 10 μM CCCP. p62 protein expression was then measured by western blotting. p62 protein was significantly decreased in p62 siRNA cells under basal conditions and following CCCP treatment, when compared to scram cells. **p <* *0.01 versus scram vehicle or scram CCCP;* n* = 3. (e) Mitochondrial content was measured in SH‐SY5Y cells treated with scram or p62 siRNA for 72 h by measuring citrate synthase (CS) activity. For the last 24 h, cells were treated with vehicle or 10 μM CCCP and data are expressed as % of vehicle. *p <* *0.05 versus scram CCCP treatment; *n* = 5.

To confirm that increased p62 expression was a response to PINK1/parkin‐mediated mitophagy and not another effect of CCCP, we investigated p62 expression in SH‐SY5Y cells constitutively expressing either a control scrambled shRNA plasmid (scram) or shRNA plasmid with a PINK1 target sequence. PINK1 mRNA levels in two PINK1 KD clonal cell lines were decreased by 67% and 72% (scram, 0.0050 ± 0.0004 relative mRNA expression; PINK1 118‐3, 0.0011 ± 0.0001 relative mRNA expression; PINK1 118‐4, 0.0017 ± 0.0011 relative mRNA expression). These two PINK1 KD cell lines did not exhibit accumulation of full‐length PINK1 upon CCCP treatment, unlike scram cells or a PINK1 KD cell line with only a 45% decrease in PINK1 mRNA levels (117‐2; Fig. 3a). Cleaved forms of PINK1 were not detected as FL‐PINK1 cannot be imported in to depolarised mitochondria and therefore is not cleaved by mitochondrial proteases such as PARL (Jin *et al*. 2010; Matsuda *et al*. 2010). Since full‐length PINK1 does not accumulate on mitochondria, this will inhibit parkin recruitment to mitochondria, and thus mitophagy (Matsuda *et al*. 2010; Narendra *et al*. 2010b; Vives‐Bauza *et al*. 2010). This was reflected when we measured the loss of mitochondrial content in these cells following 24 h CCCP treatment by assaying the activity of the mitochondrial enzyme CS. CS activity was decreased by 33% in scram cells treated with CCCP for 24 h (67.3 ± 4.0% relative to vehicle‐treated cells), while activity was only decreased by 22% in PINK1 KD cells (77.9 ± 1.8% relative to vehicle‐treated cells), and was significantly different compared to scram cells (*p *<* *0.05; *n* = 4). CS is a quantitative measurement of mitochondrial content, and we have previously shown that loss of CS activity following CCCP treatment can be (a) increased with over‐expression of parkin or (b) impaired by transient KD of PINK1 with siRNA (Gegg *et al*. 2010), and therefore can be used as a measure of mitophagy.

p62 protein levels were increased by 7.3‐fold in scram cells following 18 h of CCCP treatment (Fig. 3b). However, p62 levels were only increased by 1.9‐fold in the PINK1 KD cells with impaired mitophagy (clone 118‐3), and was significantly lower when compared to scram cells treated with CCCP (*p *<* *0.05). The other PINK1 KD clone (118‐4) resulted in 1.8 ± 0.1‐fold induction of p62 protein levels.

When starvation‐induced macroautophagy was induced in SH‐SY5Y cells, p62 protein levels were significantly decreased after both 6 and 24 h (Fig 3c). Note that starvation of SH‐SY5Y cells for 24 h had no effect on mitochondrial content, as measured by CS activity (control, 498 ± 24 nmol/min/mg protein; starvation, 529 ± 27 nmol/min/mg protein).

All the above data suggest that induction of p62 expression in SH‐SY5Y cells following CCCP is related to activation of mitophagy rather than a generic response to induction of macroautophagy or another effect of CCCP. An explanation for this p62 induction might be to help maintain the rate of mitophagy. Similar to reports on other cells like HEK293, HeLa, cardiomyocytes and mouse embryonic fibroblasts (MEFs) (Ding *et al*. 2010; Geisler *et al*. 2010), the presence of p62 is required for mitophagy in SH‐SY5Y cells. KD of p62 protein levels by 79% (*p *<* *0.01; Fig. 3d) in SH‐SY5Y cells significantly reduced mitophagy (Fig. 3e). CCCP treatment of SH‐SY5Y cells transfected with scrambled siRNA resulted in a 37% decrease in mitochondrial content after 18 h (as measured by CS activity). Mitochondrial content was only decreased by 16% in cells with p62 KD, and was significantly less than cells transfected with scrambled siRNA (*p *<* *0.05).

### Nrf2 and TFEB translocate to nucleus following CCCP treatment and affects transcription of p62

**Figure 4:**
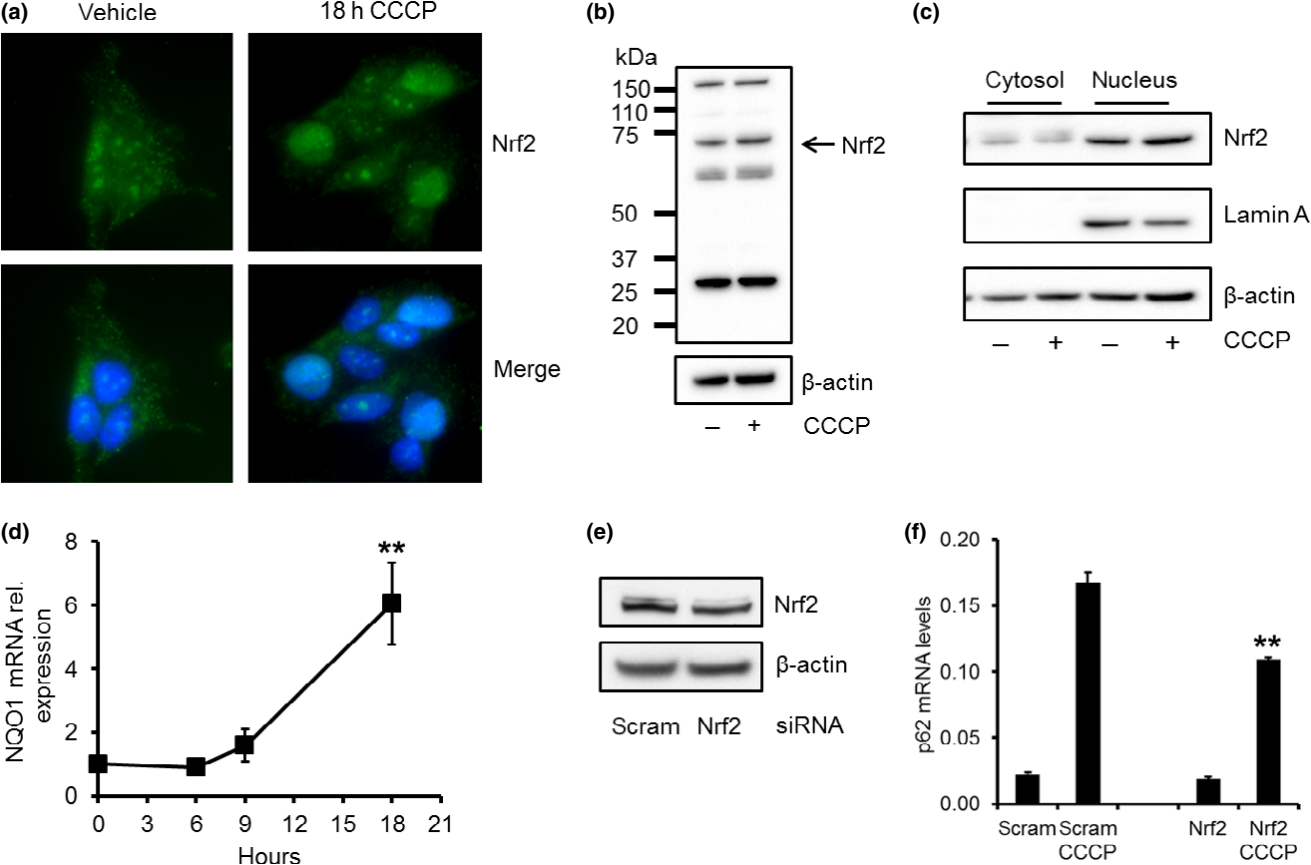
Nrf2 translocates to nucleus and affects p62 expression following carbonyl cyanide m‐chlorophenylhydrazone (CCCP) treatment. (a) SH‐SY5Y cells were treated with 10 μM CCCP for 18 h and Nrf2 nuclear localisation measured by immunofluorescence. Nrf2 is in green; nuclei in blue (4′,6‐diamidino‐2‐phenylindole stain). (b) Nrf2 protein levels measured by western blotting were also increased following 18 h of CCCP treatment. (c) SH‐SY5Y cells were treated with 10 μM CCCP for 18 h and cytosolic and nuclear fractions prepared. Nrf2 protein was detected in cytosolic (0.6% of total volume) and nuclear fractions (18% of total volume) by western blotting. The purity of the nuclear and cytosolic fractions was assessed by western blotting using lamin A and β‐actin antibodies respectively. (d) RNA was extracted from SH‐SY5Y cells treated with CCCP for 0–18 h and qPCR performed for NQO1 mRNA levels. Data normalised against β‐actin mRNA. **p <* *0.01 versus 0 h; *n* = 3. (e) SH‐SY5Y cells were treated with scram or Nrf2 siRNA for 72 h. Knockdown (KD) of Nrf2 protein levels was measured by western blotting. (f) For the last 24 h, sister wells were treated with vehicle or 10 μM CCCP. RNA was extracted and p62 mRNA levels measured by qPCR. Nrf2 knockdown significantly decreased p62 mRNA levels after CCCP treatment. Data normalised against β‐actin mRNA. **p <* *0.01 versus scram CCCP; *n* = 3.

Two transcription factors that are known to regulate the expression of p62 are Nrf2 and TFEB (Jain *et al*. 2010; Settembre *et al*. 2012). When SH‐SY5Y cells were treated with CCCP for 18 h the nuclear localisation of Nrf2 was found to be increased by immunofluorescence (Fig. 4a). A modest significant increase in total Nrf2 protein expression was observed during this period (116 ± 5% relative to vehicle‐treated SH‐SY5Y; *p *<* *0.05; *n* = 6; Fig. 4b). We are confident that this is the correct band for Nrf2 as it is corresponds to the predicted molecular weight of 68 kDa and this is diminished by treatment of cells with Nrf2 siRNA (see below). Nuclear and cytosol fractionation of SH‐SY5Y cells treated with CCCP for 18 h supported the immunofluorescence data (Fig. 4c). Nrf2 localised to the nucleus was significantly increased in cells treated with CCCP for 18 h, when expressed against the nuclear protein lamin A (vehicle, 0.82 ± 0.14 Nrf2/lamin A; CCCP, 1.33 ± 0.23; *p *<* *0.05; *n* = 4). Please note the Nrf2 signal in the cytosol is much stronger. Therefore, only a small proportion of the cytosolic fraction (0.6% of total) is loaded so that the nuclear fraction of Nrf2 is more easily detected. The increased nuclear location of Nrf2 coincided with a significant increase in the transcription of NQO1 (Fig. 4d), a well described Nrf2 target gene (Kwon *et al*. 2012; Ichimura *et al*. 2013). These data all suggest that Nrf2 does translocate to the nucleus following CCCP treatment.

To determine whether nuclear translocation of Nrf2 also affected p62 transcription, we knocked down Nrf2 with siRNA for 72 h. Quantification of western blots for Nrf2 indicated that protein levels were significantly decreased by 38% in Nrf2 KD cells (*p *<* *0.05; *n* = 4), when compared to scram cells (Fig. 4e). When these cells were treated with CCCP for 24 h, the induction of p62 mRNA was significantly decreased by 35% (*p *<* *0.01; *n* = 3) when compared to scram cells treated with CCCP (Fig. 4f).

**Figure 5:**
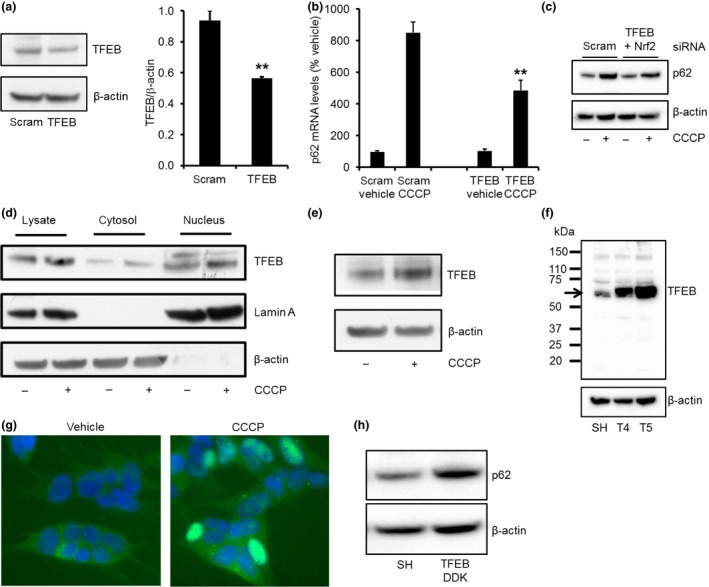
TFEB translocates to the nucleus and affects p62 expression following carbonyl cyanide m‐chlorophenylhydrazone (CCCP) treatment. (a) SH‐SY5Y cells were treated with scrambled (scram) or TFEB siRNA for 72 h and TFEB protein expression measured by western blotting. **p <* *0.01 versus scram; *n* = 4. (b) During the last 24 h of TFEB knock‐down, cells were treated with vehicle or 10 μM CCCP for 24 h and RNA extracted. p62 mRNA levels were measured by qPCR and normalised to β‐actin mRNA levels. **p <* *0.01 versus scram CCCP‐treated cells; *n* = 4. (c) p62 protein levels were also measured following 24 h CCCP treatment of SH‐SY5Y cells treated with scram or TFEB and Nrf2 siRNA for previous 72 h. (d) Following 24 h CCCP treatment of SH‐SY5Y cells, total cell lysates were prepared and subcellular fractionation performed to generate cytosolic and nuclear fractions. TFEB protein was detected by western blotting. Lamin A and β‐actin were also measured to assess purity of the nuclear and cytosolic fractions respectively. (e) SH‐SY5Y cells were treated with CCCP for 24 h and endogenous TFEB measured in total cell lysates by western blotting. (f) SH‐SY5Y cell lines expressing exogenous TFEB with a DDK tag (clones T4 and T5) were generated. TFEB protein expression in parental SH‐SY5Y cell line and clones T4 and T5 was measured by western blotting with a TFEB antibody. Endogenous TFEB is shown by arrow. (g) TFEB‐DDK cells were treated with vehicle or CCCP for 24 h and TFEB detected by immunofluorescence with an antibody against DDK (green). Nuclei are stained blue with 4′,6‐diamidino‐2‐phenylindole. (h) Total cell lysates were prepared from SH‐SY5Y cells (SH) and TFEB‐DDK cells and p62 protein levels measured by western blotting.

When TFEB protein levels were significantly knocked down in SH‐SY5Y cells following 72 h of treatment with siRNA (Fig. 5a), the increase in p62 mRNA levels were also significantly lower (*p *<* *0.01; *n* = 4) following 24 h CCCP treatment, when compared to cells treated with scram siRNA and CCCP (Fig. 5b). Since we have shown that increased expression of p62 mRNA following CCCP treatment was significantly impaired following Nrf2 or TFEB KD, we transiently transfected SH‐SY5Y cells with both TFEB and Nrf2 and measured the effect of CCCP on p62 protein expression (Fig 5c). Following 24 h of CCCP treatment, p62 protein levels were increased by 2.07 ± 0.21 fold in scram treated cells (*n* = 4). In the TFEB and Nrf2 KD cells, p62 protein expression was lower (1.46 ± 0.28; *n* = 4), although not significantly. This may be explained by the recent report that homologues of TFEB, MITF and TFE3, are also activated following PINK1/parkin‐mediated mitophagy, and may contribute to the expression of p62 (Nezich *et al*. 2015). Next, we confirmed that TFEB translocated to the nucleus of SH‐SY5Y cells after CCCP treatment. Subcellular fractionation of SH‐SY5Y cells treated with CCCP followed by western blotting of cytosolic and nuclear fractions indicated that the proportion of endogenous TFEB in the nucleus was increased (Fig. 5d; SH + vehicle, 0.84 ± 0.01 TFEB/lamin A; SH + CCCP, 2.38 ± 0.68 TFEB/lamin A; *p *<* *0.01; *n* = 5). Nuclear TFEB can also increase its own expression by a feed‐forward mechanism (Sardiello *et al*. 2009; Settembre *et al*. 2013). TFEB mRNA levels were significantly increased by 2.11 ± 0.38 fold (*p *<* *0.05; *n* = 4) following treatment with CCCP for 18 h (TFEB mRNA levels were unchanged after 6 or 9 h of CCCP treatment). This was coincident with a significant twofold (*p *<* *0.01; *n* = 6) increase in TFEB protein expression after 24 h of CCCP treatment (Fig. 5e).

Detection of endogenous TFEB by immunofluorescence was unsuccessful. SH‐SY5Y cells expressing recombinant human TFEB with a DDK tag (TFEB‐DDK; Fig. 5f) were generated, and translocation of TFEB‐DDK to the nucleus was detected 24 h after CCCP treatment (Fig. 5g). Note that p62 protein levels in TFEB‐DDK cells were significantly increased 163% (Fig. 5h), when compared to the parental SH‐SY5Y cell line (SH‐SY5Y, 0.347 ± 0.088 p62/β‐actin; TFEB‐DDK, 0.568 ± 0.109; *p *<* *0.01; *n* = 5). These data suggest that TFEB does translocate to the nucleus following CCCP treatment and that this has an effect on p62 mRNA levels.

### TFEB increases lysosomal biogenesis following CCCP‐induced mitophagy

**Figure 6:**
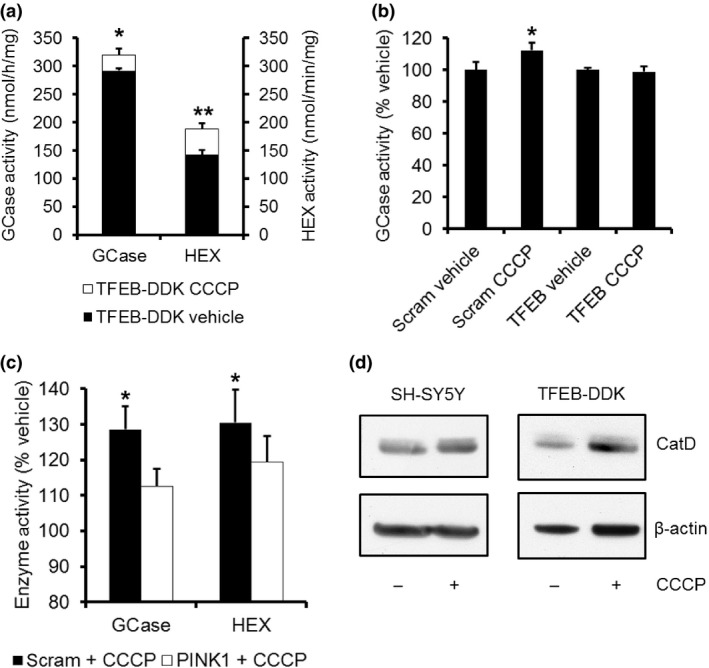
Lysosomal enzyme activity increased following carbonyl cyanide m‐chlorophenylhydrazone (CCCP)‐induced mitophagy. (a) TFEB‐DDK cells were treated with vehicle (black) or 10 μM CCCP (white) for 24 h and the activities of GCase (nmol/h/mg protein) and β‐hexosaminidase (HEX) (nmol/min/mg protein) were measured in cell lysates. *p <* *0.05 versus vehicle; **p <* *0.01 versus vehicle; *n* = 4. (b) SH‐SY5Y cells treated with scrambled (scram) or TFEB siRNA for 72 h. For the last 24 h cells were treated with vehicle or 10 μM CCCP and GCase activity measured in cell lysates. *p <* *0.05 versus vehicle; *n* = 4. (c) SH‐SY5Y cells expressing scrambled (scram) shRNA or PINK1 shRNA were treated with vehicle or CCCP for 24 h. GCase and HEX enzyme activity was measured in cell lysates. *p <* *0.05 versus vehicle; *n* = 5. (d) SH‐SY5Y cells or TFEB‐DDK cells were treated with vehicle or CCCP for 24 h and cathepsin D protein levels measured by western blotting.

In addition to regulating the transcription of p62, TFEB has been reported to be a master regulator of lysosomal biogenesis (Sardiello *et al*. 2009). One of the genes that TFEB regulates is *GBA1*, which encodes for the lysosomal enzyme GCase (EC 3.2.1.45). Mutations in the *GBA1* gene increase the risk of developing PD and loss of GCase activity has been reported in sporadic PD brains (Sidransky *et al*. 2009; Gegg *et al*. 2012). Loss of GCase activity has been implicated in impairment of the ALP, including mitophagy (Mazzulli *et al*. 2011; Sardi *et al*. 2011; Osellame *et al*. 2013). Treatment of SH‐SY5Y cells with CCCP for 24 h increased *GBA1* mRNA levels 2.45‐fold compared to vehicle‐treated cells (SH‐SY5Y + vehicle, 100 ± 11.0%; SH‐SY5Y + CCCP, 245.3 ± 74.8%; *p *=* *0.05, *n* = 4). The activities of GCase and another TFEB‐regulated lysosomal enzyme HEX (EC 3.2.1.52) were significantly increased in SH‐SY5Y cells by 108.8 ± 1.7% (*p *<* *0.01, *n* = 6) and 123.9 ± 3.6% (*p *<* *0.05, *n* = 5), respectively, following 24 h of CCCP treatment. The activities of GCase and HEX were significantly increased by 109.7% and 132.2%, respectively, in TFEB‐DDK expressing SH‐SY5Y cells treated with CCCP for 24 h (Fig. 6a). Furthermore, the CCCP‐induced increase in GCase activity was not observed in SH‐SY5Y cells with TFEB KD (Fig. 6b).

To determine whether the increase in GCase and HEX activities were a result of mitophagy induction or another effect of CCCP, lysosomal activity was measured in scram or PINK1 KD SH‐SY5Y cells. The activities of both GCase and HEX were significantly increased (*p *<* *0.05) in scram SH‐SY5Y cells treated with CCCP by 128.6 ± 6.4% and 130.5 ± 9.3%, respectively, when compared to vehicle‐treated scram cells (Fig. 6c). CCCP treatment of PINK1 KD cells did not significantly increase either GCase (112.5 ± 5.0%) or HEX (119.4 ± 7.2%).

Protein expression of the lysosomal protease cathepsin D (28 kDa mature form; EC 3.4.23.5) was also significantly increased by CCCP treatment in SH‐SY5Y cells (Fig. 6d; SH‐SY5Y + vehicle, 0.75 ± 0.08 catD/actin; SH‐SY5Y +CCCP, 0.99 ± 0.10 catD/actin; *p *<* *0.05; *n* = 6) or TFEB‐DDK‐expressing cells (TFEB‐DDK + vehicle, 0.55 ± 0.11 catD/actin; TFEB‐DDK + CCCP, 0.73 ± 0.13 catD/actin; *p *<* *0.05; *n* = 4).

### Increased TFEB expression increases mitochondrial content in SH‐SY5Y cells

**Figure 7:**
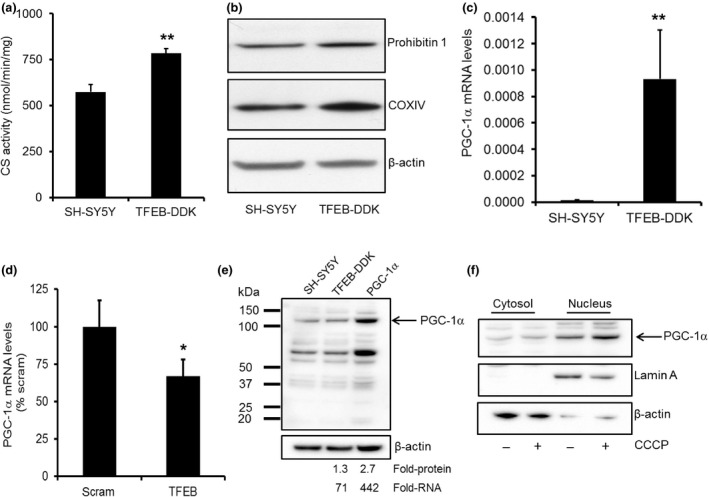
Mitochondrial content and PGC‐1α levels are increased in cells with greater TFEB protein expression. (a) Mitochondrial content was measured in SH‐SY5Y cells and TFEB‐DDK cells under basal conditions by assaying citrate synthase (CS) activity. **p <* *0.01 versus SH‐SY5Y; *n* = 6. (b) Western blotting for the mitochondrial proteins prohibitin 1 and cytochrome oxidase (COX) subunit IV also indicated that mitochondrial content was increased in TFEB‐DDK cells. (c) PGC‐1α mRNA levels were measured in SH‐SY5Y cells and TFEB‐DDK cells under basal conditions by qPCR. Data normalised against β‐actin mRNA levels. **p <* *0.01 versus SH‐SY5Y cells; *n* = 3. (d) SH‐SY5Y cells were treated with scrambled (scram) or TFEB siRNA for 72 h and PGC‐1α mRNA levels measured. *p <* *0.05 versus scram; *n* = 4. (e) PGC‐1α protein levels were detected by western blotting in total cell lysates of SH‐SY5Y cells, TFEB‐DDK cells and a SH‐SY5Y cell line over‐expressing human PGC‐1α. The fold increase in PGC‐1α protein density for this blot and PGC‐1α mRNA levels of the respective cell lines are reported underneath the blot. (f) TFEB‐DDK cells were treated with 10 μM carbonyl cyanide m‐chlorophenylhydrazone (CCCP) for 18 h and cytosolic and nuclear fractions prepared. PGC‐1α protein was detected in cytosolic (3.5% of total volume) and nuclear fractions (35% of total volume) by western blotting. The purity of the nuclear and cytosolic fractions was assessed by western blotting using lamin A and β‐actin antibodies respectively.

TFEB has been shown to act in the same pathway as PGC‐1α in the clearance of aggregated huntingtin and lipid catabolism (Tsunemi *et al*. 2012; Settembre *et al*. 2013). Since PGC‐1α plays a role in mitochondrial biogenesis, we hypothesised that TFEB may also translocate to the nucleus following mitophagy induction to help stimulate synthesis of new mitochondria via PGC‐1α. First, we investigated mitochondrial content in SH‐SY5Y cells over‐expressing TFEB‐DDK (T5 clone). CS activity was significantly increased by 36% (*p *<* *0.01; *n* = 6) in TFEB‐DDK cells (Fig. 7a). The mitochondrial proteins COXIV and prohibitin 1 were also increased when assessed by western blotting (Fig. 7b) by 150 ± 33% and 161 ± 36% (*p *<* *0.05 vs. SH‐SY5Y cells; *n* = 4) respectively. CS activity (137 ± 18%) and the expression of COXIV (208.1 ± 48.9%) and prohibitin 1 (209.1 ± 23.9%) protein were all increased in another SH‐SY5Y cell line over‐expressing TFEB‐DDK (denoted T4; *p *<* *0.05 vs. SH‐SY5Y cells; *n* = 6–7). These data suggest that cells with increased TFEB expression have significantly more mitochondria.

This increase in mitochondrial content was accompanied by a significant (*p *<* *0.01) increase in PGC‐1α mRNA levels (Fig. 7c). Conversely, TFEB KD in normal SH‐SY5Y cells resulted in a significant 33% decrease in PGC‐1α mRNA levels (*p *<* *0.05; Fig. 7d). Endogenous PGC‐1α protein is difficult to detect, with a half‐life of <30 min (Olson *et al*. 2008; Adamovich *et al*. 2013). We have measured PGC‐1α protein levels in SH‐SY5Y and have used a SH‐SY5Y cell line constitutively over‐expressing human PGC‐1α to validate the antibody (Fig. 7e). A band of the correct size (110 kDa) was detected in SH‐SY5Y cells. However, despite PGC‐1α mRNA levels being increased 71‐fold in TFEB‐DDK cells (Fig 7c), only a slight increase in protein expression was detected (135 ± 20%; *n* = 8). Even when PGC‐1α mRNA was increased by 442‐fold in PGC‐1α over‐expressing cells, protein expression was only increased by 267% (Fig. 7e). While an increase in PGC‐1α protein levels was difficult to detect in TFEB‐DDK cells, there was evidence that upon 18 h of CCCP treatment, 2.1 ± 0.4‐fold more PGC‐1α was detected in the nucleus, when compared to vehicle treatment (Fig. 7f; *n* = 3), suggesting PGC‐1α is activated upon mitophagy induction.

## Discussion

**Figure 8:**
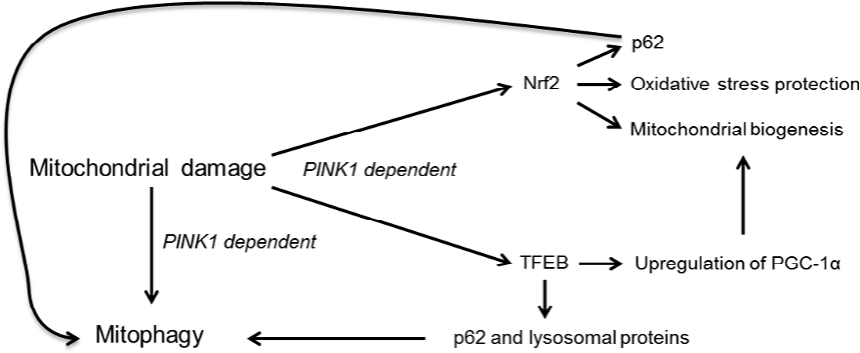
Activation of Nrf2 and TFEB following mitophagy induction. Stimulation of PINK1‐dependent mitophagy results in the nuclear translocation of the transcription factors Nrf2 and TFEB. Both transcription factors increase the expression of the autophagic protein p62. Nrf2 is involved in the antioxidant response and mitochondrial biogenesis. TFEB also increases lysosomal biogenesis and can affect mitochondrial biogenesis by increasing expression of PGC‐1α. Activation of Nrf2 and TFEB following damage to mitochondria and induction of mitophagy will likely coordinate protection from oxidative stress (via antioxidant response element), replace damaged mitochondria removed by mitophagy, and maintain efficient macroautophagy by stimulating expression of autophagic proteins such as p62 and synthesis of lysosomal enzymes.

In this report, we show that there is a significant increase in the synthesis of the autophagic protein p62 via the transcription factors Nrf2 and TFEB following activation of PINK1/parkin‐mediated mitophagy. TFEB also increased the expression of lysosomal enzymes such as GCase, HEX and cathepsin D. Furthermore, TFEB was found to modulate PGC‐1α and increase the mitochondrial content of cells. Therefore, activation of TFEB following induction of mitophagy may play a role in the biogenesis of mitochondria as well as lysosomes (Fig. 8).

The increase in p62 mRNA and protein levels is likely a cellular response to replace the autophagic protein as it is degraded with damaged mitochondria. The increase in p62 protein levels was impaired in PINK1‐deficient SH‐SY5Y cells supporting the idea that this increase was related to mitophagy induction. Furthermore, we found that the expression of p62 was not increased in SH‐SY5Y cells following starvation‐induced macroautophagy. A large number of mitochondrial proteins are ubiquitinated following CCCP‐induced mitophagy (Gegg *et al*. 2010; Matsuda *et al*. 2010; Sarraf *et al*. 2013), some of which are likely to be bound by p62/LC3 to aid in the recruitment of mitochondria to APs and localisation to perinuclear clusters (Narendra *et al*. 2010a; Okatsu *et al*. 2010). Similar to previous reports, we found that p62 was necessary for mitophagy to occur (Ding *et al*. 2010; Geisler *et al*. 2010). An increase in p62 protein levels has also been found in HeLa cells (Sarraf *et al*. 2013) and MEFs (Ichimura *et al*. 2013) following treatment with depolarising agents CCCP and valinomycin respectively. An increase in autophagy proteins ATG5, ATG12 and LC3B has also been noted in HUVECs following induction of mitophagy by DNA damage, although the authors did not investigate p62 (Mai *et al*. 2012). During the submission process of this paper, TFEB and two other homologues (MITF and TFE3) from the same transcription factor family were reported to be activated in HeLa cells following mitophagy induction in a PINK1‐ and parkin‐dependent manner (Nezich *et al*. 2015). The co‐activation of MITF and TFE3 most likely explains why the KD of Nrf2 and TFEB (either on their own or in combination) did not completely abolish the increase in p62 mRNA and protein levels following mitophagy induction.

While the increase in p62 might be because of a general up‐regulation of the ALP, it cannot be discounted that the elevation of p62 is for another purpose. In addition to its role in autophagy, p62 has been shown to regulate the oxidative stress response and mitochondrial biogenesis via Nrf2 (Jain *et al*. 2010; Kwon *et al*. 2012; Ichimura *et al*. 2013). Under basal conditions, Nrf2 is constitutively degraded by the ubiquitin–proteasome system via its association with KEAP1, an adaptor for a cullin‐ring ubiquitin ligase complex. Upon oxidative stress, this complex dissociates allowing Nrf2 to translocate to the nucleus and induce transcription of genes with an antioxidant response element, including NQO1 and p62. It is of interest that Nrf2 and p62 exist in a positive feedback loop; p62 disrupts the Nrf2‐KEAP1 complex by direct interaction resulting in stabilisation of Nrf2 (Jain *et al*. 2010; Ichimura *et al*. 2013). Therefore, the activation of Nrf2 we observe after CCCP treatment may in part be mediated via p62, increasing mitochondrial biogenesis and antioxidant defences (Fig. 8). Intriguingly, p62 knockout MEFs exhibit increased oxidative stress, fragmentation of mitochondria, and decreased mtDNA levels (Okatsu *et al*. 2010; Kwon *et al*. 2012; Seibenhener *et al*. 2013). Whether these observations are a result of impaired antioxidant response and/or mitophagy remains unclear.

TFEB coordinates the expression of lysosomal and autophagic proteins following starvation‐induced macroautophagy (Settembre *et al*. 2012). We report that the induction of mitophagy also increased the nuclear localisation of TFEB and expression of TFEB target genes such as *GBA1* and *HEXB* following mitophagy induction. We hypothesise that this is required to ensure prolonged activation of the ALP during mitophagy. GCase activity was only increased by approximately 10% after 24 h of CCCP treatment. Longer CCCP treatment results in cell death (after 30 h), so it is unknown if GCase activity was increased further at later time points. Since the half‐life of GCase has been estimated to be about 30 h (Witte *et al*. 2010), and is thus relatively long lived, perhaps induction does not need to be so great. Indeed the induction of HEXB mRNA levels was reported to be greater than GBA mRNA levels in HeLa cells over‐expressing TFEB (Sardiello *et al*. 2009).

It is becoming increasingly evident that the functions of TFEB and PGC‐1α are interconnected (Tsunemi *et al*. 2012; Settembre *et al*. 2013). The KD of TFEB has been shown to prevent the PGC‐1α‐mediated reversal of huntingtin aggregation (Tsunemi *et al*. 2012). The authors showed that PGC‐1α bound to a TFEB‐luciferase reporter construct suggesting PGC‐1α was upstream of TFEB. Conversely, the TFEB‐regulation of lipid metabolism in the liver was mediated via the transcription of several genes, including PGC‐1α (Settembre *et al*. 2013). Therefore, activation of TFEB might also contribute to the increased mitochondrial biogenesis observed after PINK1/parkin‐mediated mitophagy. The expression of two mitochondrial proteins (prohibitin 1 and COXIV) was significantly increased in SH‐SY5Y cell lines expressing exogenous TFEB. This was coincident with a significant increase in the expression of PGC‐1α mRNA. Induction of mitophagy with CCCP in TFEB‐DDK cells also increased the nuclear localisation of PGC‐1α. A coordinated up‐regulation of TFEB and PGC‐1α has recently been reported in a knock‐out model of GCN5L1, a component of the mitochondrial deacetylase machinery (Scott *et al*. 2014). KD of GCN5L1 in liver cells increased the co‐localisation of mitochondria with LC3, p62 and ubiquitin in a parkin‐independent manner (Webster *et al*. 2013). However, analysis in GCN5L1 knock‐out MEFs indicated that while TFEB‐mediated autophagy was activated, there was no loss of mitochondrial content since the expression of PGC‐1α was also increased, thus balancing mitophagy with biogenesis (Scott *et al*. 2014).

The pathway(s) by which TFEB and PGC‐1α are activated and how they are coordinated remains to be elucidated. CCCP treatment has previously been shown to increase the transcription of PINK1 in a calcium‐dependent manner by an unknown transcription factor (Gómez‐Sánchez *et al*. 2014). CCCP treatment dysregulates calcium homeostasis in SH‐SY5Y cells and thus may induce PGC‐1α via the calcium‐sensitive cAMP response element binding protein (Zhu *et al*. 2013; Gómez‐Sánchez *et al*. 2014). However, the increased expression of p62 and lysosomal proteins was diminished in PINK1‐deficient cells despite the presence of CCCP, suggesting that transcription factor activation is a mitophagy‐specific response, rather than a generalised increased in cellular calcium. Further work is required to determine how TFEB is activated. TFEB can be phosphorylated by kinases such as mTORC1 and protein kinase Cβ (Martina *et al*. 2012; Roczniak‐Ferguson *et al*. 2012; Settembre *et al*. 2012; Ferron *et al*. 2013), with phosphorylation of serines being implicated in both preventing nuclear translocation of TFEB (Martina *et al*. 2012; Roczniak‐Ferguson *et al*. 2012) and being required for the biological functions of TFEB (Peña‐Llopis *et al*. 2011; Ferron *et al*. 2013). Since PINK1 is a serine/threonine kinase, it is a putative candidate for directly phosphorylating TFEB or initiating a kinase cascade to activate TFEB. Nezich *et al*. 2015 were also unable to exactly identify how nuclear translocation of TFEB is achieved following induction of PINK1/parkin‐mediated mitophagy in HeLa, but did rule out the possible involvement of 5′ AMP‐activated protein kinase, which can inhibit mTORC1 (Lamb *et al*. 2013). However, data suggested that parkin, presumably following phosphorylation by PINK1, inactivated Rag GTPases (Nezich *et al*. 2015). Inactivation of Rag GTPases has been shown to cause mTORC1 to dissociate from TFEB, thus decreasing the phosphorylation of TFEB and allowing translocation to the nucleus (Settembre *et al*. 2012).

There is increasing interest in TFEB and neurodegenerative disorders associated with impaired proteostasis. In addition to the link between TFEB and huntingtin (Tsunemi *et al*. 2012), TFEB‐mediated autophagy has been reported to protect against α‐synuclein‐induced dopaminergic neuronal loss (Decressac *et al*. 2013). The expression of lysosomal proteins such as LAMP2A and GCase are decreased in the brains of sporadic PD brains (Alvarez‐Erviti *et al*. 2010; Dehay *et al*. 2010; Gegg *et al*. 2012). These deficiencies very likely contribute to the impairment of the ALP and thus the protein aggregation and mitochondrial dysfunction associated with PD (Terman *et al*. 2010; Nixon 2013). Our study suggests that the activation of TFEB might not only be a strategy to improve lysosomal function, and hence autophagy, but would also be useful in promoting the synthesis of functional mitochondria.
